# Myositis Ossificans in Short External Rotator Muscles of the Hip: Temporal Changes on CT

**DOI:** 10.7759/cureus.93983

**Published:** 2025-10-06

**Authors:** Chiaki Sato, Akio Fujii, Ryo Hidaka, Masaru Kamitani, Kenta Matsuda, Hiroshi Oba, Asako Yamamoto

**Affiliations:** 1 Radiology, Teikyo University School of Medicine, Tokyo, JPN; 2 Orthopaedic Surgery, Teikyo University School of Medicine, Tokyo, JPN

**Keywords:** cerebrovascular disease, ct, hip, myositis ossificans, short external rotator muscles

## Abstract

Introduction

Swelling in the short external rotator muscles (SERMs) in patients with cerebrovascular disease may regress to non-traumatic myositis ossificans. This study investigates the temporal changes in CT findings of myositis ossificans, aiming to provide exploratory insights for diagnostic accuracy and clinical management.

Methods

We retrospectively reviewed 1,469 CT examinations of the pelvis conducted at our institution between 2001 and 2023, totaling 436 cases of cerebrovascular disease. We selected 20 patients showing swelling of SERMs, including six patients with bilateral swelling. Of the 20, only patients with at least one follow-up CT, six cases (eight hip joints), were selected for analysis, including two cases with bilateral involvement. The intervals between swelling, calcification, and ossification were calculated, with calcification defined as a high-attenuation area (≥100 HU) and ossification as peripheral calcification with internal fat density.

Results

All eight hip joints exhibited calcification, either concurrently or sequentially with swelling. The mean interval from swelling to calcification was 12 ± 5.3 days, and 112 ± 102 days from swelling to ossification in six hips that developed ossification. In two hip joints, calcification disappeared without ossification developing. In all cases, the affected hip corresponded to the side of the paralyzed lower limb. On initial examination, all patients exhibited fever, two with fever of unknown origin.

Conclusion

This study determined temporal progression in CT findings of myositis ossificans in swelling of SERMs of patients with cerebrovascular disease. Further, detecting swelling, calcification, or ossification in SERMs in follow-up CT is informative in excluding infections or neoplastic conditions.

## Introduction

Myositis ossificans (MO) is a self-limiting condition characterized by ectopic ossification within skeletal muscles and can be classified as traumatic or non-traumatic [1]. Traumatic MO typically occurs in patients with a history of acute trauma and is frequently seen in sports involving repetitive microtrauma to the same site [2]. It predominantly affects young males in their 30s and commonly involves skeletal muscles of the shoulder, adductors of the hip, and the thigh extensor muscles [2].

On the other hand, non-traumatic MO occurs in the absence of any clear localized trauma and is frequently associated with indirect conditions such as cerebral or spinal injuries, involving paralysis and prolonged intensive care [3]. MO is often observed in the hip region, such as the gluteus muscles, quadriceps, and short external rotator muscles (SERMs) [3-5]. SERMs consist of six muscles: the obturator internus, obturator externus, piriformis, superior gemellus, inferior gemellus, and quadratus femoris. These muscles are located at the posterior aspect of the hip joint and contribute to the external rotation of the lower limb.

Swelling of the SERMs can be incidentally detected on body CT scans performed to investigate the source of fever in acute cerebrovascular disease patients. In such cases, differential diagnoses include infectious conditions, sarcomas, rhabdomyolysis, or myositis ossificans [6]. Among these conditions, MO is diagnosed specifically by identifying ossification formation during follow-up imaging. Furthermore, improper biopsy might cause exaggeration of conditions or pathological misdiagnosis as a soft tissue sarcoma; thus, accurate imaging interpretation is crucial [7]. Despite the clinical importance of MO, research on myositis ossificans in the SERMs remains limited [3]. While there are some studies on the radiological progression of MO based on x-ray findings of the pelvic region, there has been little research focusing on the temporal changes of MO seen on CT scans. Therefore, we conducted a single-center retrospective study to clarify the occurrence of MO in the SERMs, its prevalence, and temporal changes on CT, and to explore the appropriate timing of follow-up CT for confirming the diagnosis in patients with swollen SERMs.

## Materials and methods

**Figure 1:**
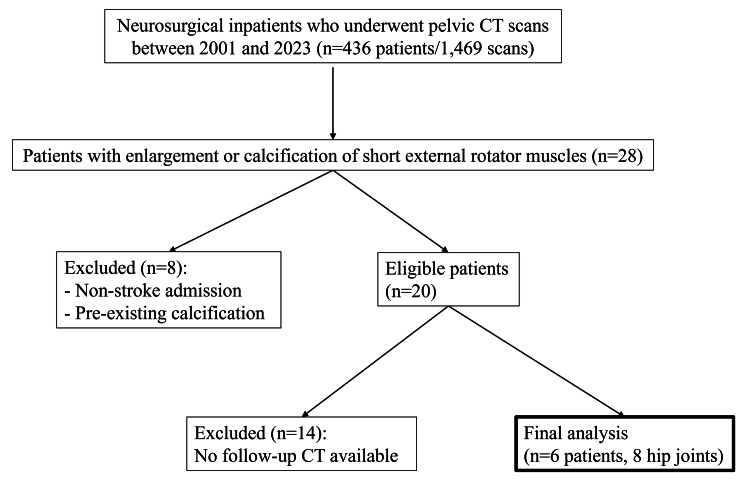
Flowchart of patient inclusion and exclusion

This retrospective study was approved by our institutional review board, which waived the need for written informed consent (No. 24-092). We retrospectively reviewed 1,469 CT examinations, which included the pelvis, of a total of 436 patients admitted to the neurosurgery department of our institution between 2001 and 2023. Cases with swelling or calcification of the SERMs were identified. Images reconstructed with a slice thickness of 5 mm and a standard kernel were assessed on axial planes in soft tissue window settings. The presence of swelling was assessed visually. Calcification was identified as positive if a small circular region of interest on a high-attenuation area showed an average value of 100 HU or more. Previous studies show that hematomas measure up to about 70 HU; we therefore set the threshold for calcification at 100 HU to exclude hematomas [8]. Ossification was defined as marginally prominent calcification (> 100 HU) accompanied by fat density within the lesion. Two radiologists (with two and 20 years of experience) independently reviewed the findings, and the results were interpreted by consensus. Patients whose hospitalization was unrelated to cerebrovascular disease or who had pre-existing calcification of the SERMs before admission were excluded. From the remaining 20 cases, we selected patients with at least one follow-up CT examination for final analysis. A flowchart of patient selection is shown in Figure 1.

The time intervals between the initial detection of swelling to calcification and to ossification were calculated. Patient records were reviewed for demographic data (age, sex). Each patient's level of consciousness, muscle strength of the affected limb (manual muscle test score), and the purpose of the initial CT were extracted from patient records dated the day of the initial CT. The purpose of follow-up CT and the presence of physical therapy during intensive care were also reviewed. Fever sources were determined based on the final diagnosis recorded in the patient records.

## Results

**Figure 2:**
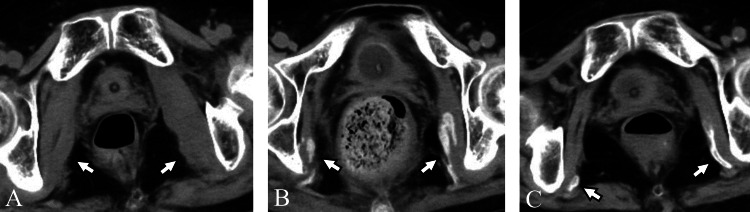
Case one A 72-year-old woman with myositis ossificans in the bilateral short external rotator muscles. (A) On the 13th day of hospitalization, CT revealed swelling in the muscles (arrows). Manual muscle testing score in both lower limbs was 0. (B) On the 27th day of hospitalization, CT performed to evaluate gastrointestinal bleeding showed calcification within the bilateral muscles (arrows), with a decrease in the swelling. The fever had resolved. (C) On the 83rd day of hospitalization, CT for investigation of the fever source revealed ossification within the bilateral muscles (arrows).

**Figure 3:**
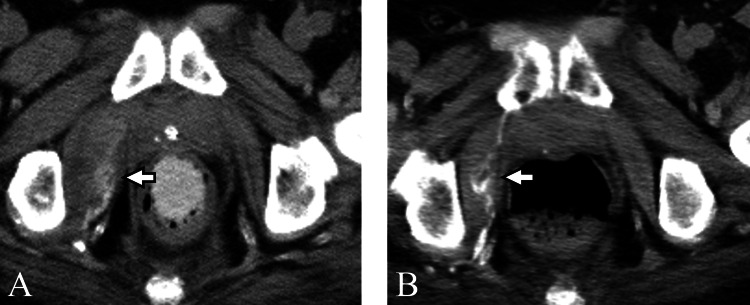
Case two A 63-year-old man with myositis ossificans in the right short external rotator muscles. (A) On the 28th day of hospitalization, CT performed for evaluation of fever demonstrated swelling accompanied by calcification in the right muscles (arrow). Manual muscle testing score in the right lower limb was 0. Pneumonia was identified as the cause of the fever. (B) On the 97th day, follow-up CT showed the development of ossification with a decrease in the swelling (arrow).

**Figure 4:**
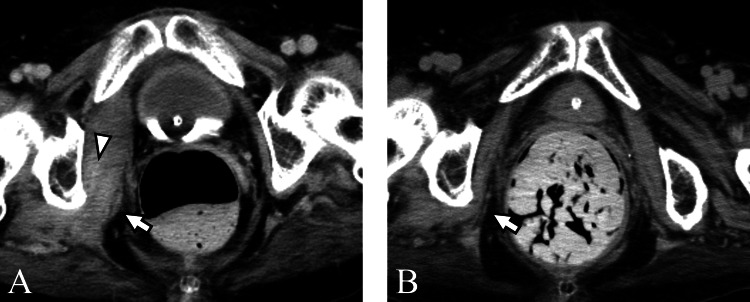
Case three A 69-year-old woman underwent a CT to evaluate fever source. (A) CT on the 18th day of hospitalization revealed swelling (arrow) and calcification (arrowhead) in the right muscles. Manual muscle testing score in the right lower limb was 0. Deep vein thrombosis was the suspected source of the fever. (B) On the 103rd day, the calcification disappeared, and no ossification was observed (arrow).

**Figure 5:**
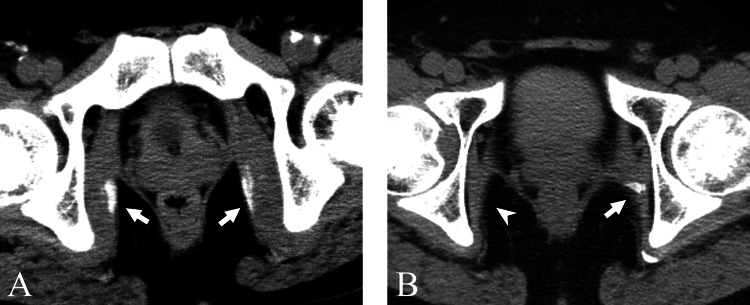
Case four A 55-year-old man with myositis ossificans in the left short external rotator muscles. (A) On the 24th day of hospitalization, CT showed swelling and calcification in the muscles bilaterally (arrows). Manual muscle testing score in both lower limbs was 2. (B) On the 360th day after admission, follow-up CT was performed due to suspected lumboperitoneal shunt failure. On the left side, ossification progression was observed (arrow). On the right side, both swelling and calcification resolved without ossification (arrowhead).

**Figure 6:**
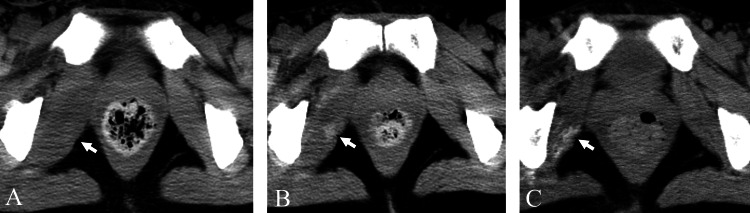
Case five A 41-year-old woman with myositis ossificans in the right short external rotator muscles. (A) On the 20th day of hospitalization, CT showed swelling of the right muscles (arrow). Manual muscle testing score in the right lower limb was 2. No other source of fever was identified. (B) On the 23rd day, CT revealed calcification within the swollen muscles (arrow). (C) On the 49th day, follow-up CT demonstrated ossification with a decrease in swelling (arrow).

**Figure 7:**
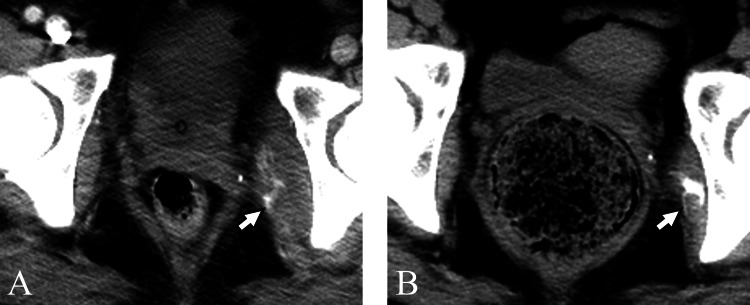
Case six A 35-year-old woman with myositis ossificans in the left short external rotator muscles. (A) CT obtained on the 36th day of hospitalization showed swelling in the left muscles (arrow). Manual muscle testing score in the left lower limb was 1. Acute pancreatitis was present as the cause of fever. (B) On the 53rd day, CT displayed calcification within the left muscles (arrow). (C) On the 136th day, CT demonstrated ossification and a decrease in swelling (arrow).

All CT scans were performed using 16 to 320-slice CT scanners. Of the 436 patients, 28 cases (6.4%) showed swelling or calcification of the SERMs during hospitalization. Of these 28 cases, 20 cases (26 hip joints; mean age 61 ± 14 years, eight males, 12 females) were identified as having lesions of the SERMs, swelling, or calcification in the first CT examination. Follow-up CT was available for six cases (eight hip joints), forming the final study group. All CT images of the six patients are presented in Figures 2-7.

Patient characteristics are summarized in Table 1. The patients ranged in age from 35 to 72 years (mean 56 ± 14 years), including two males and four females. All cases had intracranial hemorrhage as the underlying condition, accompanied by varying degrees of consciousness impairment. Physical therapy was prescribed in the intensive care unit for all six cases. All patients presented with fever, and the initial CT scan was prescribed to investigate the source of the fever in each case. The reasons for follow-up CT were for the investigation of the sources of fever (cases one and two), screening after ventriculoperitoneal shunt insertion (case three), evaluation of lumboperitoneal shunt malfunction (case four), follow-up for swelling of SERMs (case five), and follow-up for pancreatitis (case six). Specifically, to investigate suspected acute pancreatitis in one case. Subsequently, at the time of detecting swelling, all lower limbs of the affected side showed manual muscle test scores of 0-2. Of the six cases, two cases had no identifiable source of fever other than MO. The remaining four cases had the following sources of fever: pneumonia in two cases, deep vein thrombosis in one case, and acute pancreatitis in one case.

**Table 1:** Patient characteristics F: Female, GCS: Glasgow Coma Scale, M: Male, MMT: Manual Muscle Testing, MO: Myositis Ossificans, SAH: Subarachnoid Hemorrhage, SD: Standard Deviation

Case	Age	Sex	Disease	Consciousness Level at Swelling	Affected Limb MMT at Swelling	Purpose of Initial CT	Sources of Fever Other Than MO
One	72	F	Cerebellar hemorrhage	GCS: E3VTM2	1	Source of fever	None
Two	63	M	Thalamic hemorrhage	GCS: E2VTM4	0	Source of fever	Pneumonia
Three	69	F	SAH	GCS: E1VTM4	0	Source of fever	Deep vein thrombosis
Four	55	M	SAH	GCS: E3V4M6	2	Source of fever	Pneumonia
Five	41	F	Putaminal hemorrhage	GCS: E2VTM5	2	Source of fever	None
Six	35	F	Putaminal hemorrhage	GCS: E3VTM6	1	Source of fever	Acute pancreatitis
Mean ± SD	56 ± 14						

CT findings of the six cases (eight hip joints) are detailed in Table 2. Swelling of the SERMs was observed unilaterally in four cases and bilaterally in two cases (cases one and four). In all four unilateral MO cases, the affected lower limb was paralyzed. Calcification was observed in all eight hip joints either concurrently or sequentially with swelling. The time intervals from hospitalization to detection were as follows: swelling (13-36 days, mean 22 ± 7.3), calcification (18-53 days, mean 28 ± 9.9), and ossification (49-360 days, mean 135 ± 104). The interval from swelling to calcification (four hip joints) ranged from three to 17 days (mean 12 ± 5.3), and swelling to ossification (six hip joints) ranged from 29 to 336 days (mean 112 ± 102). In two hip joints, calcification resolved without progression to ossification.

**Table 2:** CT findings and time course of myositis ossificans of short external rotator muscles in six cases N/A: Not Applicable, SD: Standard Deviation

Case	Side	Swelling (day)	Calcification (day)	Ossification (day)	Swelling-to-Calcification (day)	Swelling-to-Ossification (day)
One	Right	13	27	83	14	70
	Left	13	27	83	14	70
Two	Right	28	28	97	N/A	69
Three	Right	18	18	N/A	N/A	N/A
Four	Right	24	24	N/A	N/A	N/A
	Left	24	24	360	N/A	336
Five	Right	20	23	49	3	29
Six	Left	36	53	136	17	100
Mean ± SD		22 ± 7.3	28 ± 9.9	135 ± 104	12 ± 5.3	112 ± 102

## Discussion

A range of studies has addressed the temporal progression of MO. Pathologically, muscle injury triggers an inflammatory cascade, activating endothelial cells in the muscle via cytokines and leading to the formation of multipotent mesenchymal stem cells [2]. These stem cells differentiate into myofibroblasts, chondrocytes, and osteoblasts, gradually resulting in calcification or ossification. Reflecting these pathological stages, the clinical and imaging features of MO can be divided into three phases. In the early stage following trauma, patients typically experience swelling, pain, and a restricted range of motion, but no calcification is observed [9]. Around the third week, calcification begins to appear on plain radiographs, with CT being more sensitive than x-ray in detecting early calcification [9, 10]. As the lesion matures, pain resolves within four to six weeks [3]. By approximately six weeks post-injury, ossification characterized by peripheral calcification can be detected, which may continue to evolve for up to six months or more [2].

In this retrospective study, swelling or calcification in SERMs was seen in 28/436 patients (6.4%), with MO confirmed in six patients. The interval between swelling and calcification ranged from three to 17 days (12 ± 5.3), and that between swelling and ossification ranged from 29 to 336 days (112 ± 102), consistent with the timing observed in previous studies. Based on these findings, incidental enlargement of the SERMs may suggest that a follow-up CT within two to three weeks could be useful as a rough guideline. Detecting calcification during this follow-up period may help differentiate MO from soft tissue infections or tumors. Additionally, ossification may appear as early as one month post-injury, which could support the diagnosis of MO. 

Ectopic ossification is associated with various neurological conditions [11]. Garland et al. reported a study of 496 patients with severe head injuries and concluded that heterotopic ossification was particularly associated with spasticity [12]. In this study, the hip was the most frequently affected site, and cases of ectopic ossification in the posteromedial region of the hip were associated with adductor muscle spasms. Additionally, in a group of hemiplegic patients, 70% developed heterotopic ossification on the hemiparetic side. Another study involving 48 patients with severe head injuries and impaired consciousness reported that heterotopic ossification around the joints was associated with limb paralysis and pathological muscle tone [13]. In our study, all four cases of unilateral MO occurred in SERMs on the paralyzed side. In paralyzed lower limbs, the hip tends to assume an externally rotated position due to contracture of the short rotators. When passive internal rotation is applied to the hip, it is speculated that the short rotators are overstretched, leading to microscopic muscle injuries [11]. These insights could contribute to the development of more personalized approaches to improving optimal caregiving methods and rehabilitation procedures, and programs.

In MO, cases have been reported where the lesion shrinks or disappears after ossification [14, 15]. However, in this study, we identified two hips with calcification that did not progress to ossification. Notably, in one of the two cases, the opposite hip developed MO, suggesting that while calcification is an early stage of MO, it does not always lead to ossification. The reason some cases progress to MO while others do not is still unknown, and further research is needed to clarify this.

MO is not widely recognized as a potential source of fever in paralyzed patients, which increases the likelihood that swelling in the SERMs may be overlooked, even when CT scans performed include the pelvis in the field of view. Cases of fever caused by MO have been sporadically reported [16-18]. Nuovo et al. noted that early-stage MO could be misdiagnosed as an infection and suggested that MO should be included in the differential diagnosis for culture-negative or antibiotic-resistant cases [7]. In our study, two cases demonstrated fever without any other identifiable source, suggesting that MO might have been the cause. When evaluating the source of fever in patients with lower limb paralysis using torso CT, acute-phase MO should be considered as part of the differential diagnosis.

There are several limitations to this study. First, the analysis was restricted to patients who had undergone follow-up CT examinations. As a result, the overall sample size was small, limiting the strength and generalizability of our conclusions. Second, the follow-up period was not standardized prospectively, introducing potential selection bias and resulting in a wide range of follow-up intervals and possible overestimation. In addition to the small sample size and retrospective nature, swelling was judged visually, which introduced a degree of subjectivity and potential interobserver variability despite reading by consensus. Third, the study consisted solely of inpatients admitted to the neurosurgery department, excluding patients from other specialties or outpatient care. Therefore, the findings may not reflect the full spectrum of etiologies associated with lower limb paralysis. Finally, we hypothesized that abnormal positioning and mechanical stress in paralyzed limbs might contribute to MO development; however, direct evidence is lacking in this study.

## Conclusions

This study demonstrated the time course of MO in SERMs of patients with cerebrovascular disease based on CT findings. The results showed that calcification was observed within two to three weeks after swelling, and typically progressed to ossification as early as one month later. From these findings, as a preliminary guideline, we propose a follow-up period of two or three weeks to confirm the diagnosis of early-stage MO to differentiate from infectious or neoplastic diseases. The follow-up CT will help to avoid unnecessary biopsy or treatment.

Swelling, calcification, and ossification progression occurring on the paralyzed side in our patients suggest that mechanical stress associated with immobility or abnormal posture may contribute to the onset of MO in SERMs. In addition, MO-SERMS could be a potential cause of fever, especially when no other infectious disease can be identified. Finally, this study is a retrospective study of a small number of cases. To confirm the etiology of this condition, further study is needed with a larger population and standardized follow-up intervals.
